# Exosomes of invasive urothelial carcinoma cells are characterized by a specific miRNA expression signature

**DOI:** 10.18632/oncotarget.17619

**Published:** 2017-05-04

**Authors:** Sophie Baumgart, Sebastian Hölters, Carsten-Henning Ohlmann, Rainer Bohle, Michael Stöckle, Marie Stampe Ostenfeld, Lars Dyrskjøt, Kerstin Junker, Joana Heinzelmann

**Affiliations:** ^1^ Department of Urology and Pediatric Urology, Saarland University Medical Center, 66424 Homburg, Germany; ^2^ Institute of Pathology, Saarland University Medical Center, 66424 Homburg, Germany; ^3^ Department of Molecular Medicine, Aarhus University Hospital, 8200 Aarhus, Denmark

**Keywords:** exosomes, miRNAs, bladder cancer, exosomal miRNA, tumorigenesis

## Abstract

Muscle-invasive bladder cancer (MIBC) represents a highly aggressive tumor type compared to non-muscle-invasive tumors. MIBC is characterized by specific molecular alterations, which may also modulate extracellular tumorigenic effects. Tumor-associated exosomes, especially exosomal miRNAs, are important regulators in the interaction between tumor cells and tumor microenvironment by affecting tumor-promoting processes in target cells. It is important to analyze whether their exosomal patterns also reflect the specific molecular characteristics of MIBC. The aim of this study was to compare the miRNA expression in secreted exosomes from urinary bladder cancer cells (UBC) with different degrees of invasiveness. By electron microscopy, nanotracking analysis and western blot we proofed a high quality of isolated exosomes. Microarray analysis identified an invasion-associated signature of 15 miRNAs, which is significantly altered in exosomes of invasive UBC compared to non-invasive counterparts. Therefrom, 9 miRNAs are consistent differently expressed in both, invasive cells and their secreted exosomes. The remaining 6 exosome-specific miRNAs are only deregulated in exosomes but not in their parental cells. MiRNA alterations were verified by qPCR in cell culture and urinary exosomes. In conclusion, we showed that exosomes from invasive UBC cells are characterized by a specific miRNA signature. Further analyses have to clarify the functional relevance of exosomal miRNAs secreted by invasive bladder cancer cells for modification of the tumor microenvironment and their putative role as molecular markers in liquid biopsies.

## INTRODUCTION

Urothelial bladder cancer (UBC) is the 5th most common cancer in Europe. Around 70% of patients are diagnosed with a non-muscle-invasive tumor (NMIBC) and 30% with a muscle-invasive tumor (MIBC) [1]. 50% of patients with MIBC develop distant metastases in bones, lungs and liver associated with poor prognosis [2–4]. Until now, effective curative systemic therapies are not available in metastatic stage. A better understanding of the molecular processes of tumorigenesis and progression is therefore necessary to develop more efficient anticancer treatments. Recent studies have shown that the crosstalk between tumor cells and the surrounding tissue plays a crucial role in tumorigenesis [5–8]. Besides soluble factors, secreted membrane vesicles (e.g. microvesicles, exosomes) are involved in this process by reprogramming the tumor microenvironment (TME) and generating an invasion-promoting environment [9, 10]. Exosomes are small membrane vesicles (40–100 nm), which are formed in the endosomal system and secreted by almost all cell types into the extracellular space [11]. Recipient cells can internalize exosomes receptor-mediated, either by direct membrane fusion or by phagocytosis. Therefore, they are important players in the intercellular transfer regulating cell-cell communication [12, 13]. These vesicles are characterized by specific surface markers, such as CD81, CD63, syntenin or Alix, and contain also different molecules including proteins, DNA, mRNA and miRNA. The molecular content of exosomes partly reflects the molecular composition of the parental cells [14, 15]. By transferring their content to recipient cells, exosomes regulate not only physiological processes such as tissue repair or blood coagulation but also pathological processes like impaired wound healing, tumor development or formation of a premetastatic niche [16]. In particular, exosomal miRNAs play an important role in the interaction between tumor cells and TME. MiRNAs are incorporated into exosomes at high levels and can quickly and effectively affect major tumor-supporting mechanisms in targeted cells, such as invasion, proliferation, differentiation and migration [17, 18]. Exosomal miRNAs are linked to tumorigenesis and invasiveness and are possible key players in cell-cell communication [19]. So far only few studies investigated the role of tumor-associated exosomes in development and progression of UBC. It is known that invasive UBC are characterized by specific molecular alterations including altered miRNA expression, so it would be important to analyze whether their exosomes are also reflecting this aggressive tumor type [20, 21]. Therefore, the aim of the study was the comparison of the miRNA expression pattern of secreted exosomes in correlation with the invasiveness of UBC cells *in vitro* and *in vivo*. To the best of our knowledge, we present the first data indicating that exosomes secreted by invasive UBC cells are characterized by a specific miRNA expression pattern.

## RESULTS

### Invasive UBC cells are characterized by a specific miRNA expression pattern

**Figure 1:**
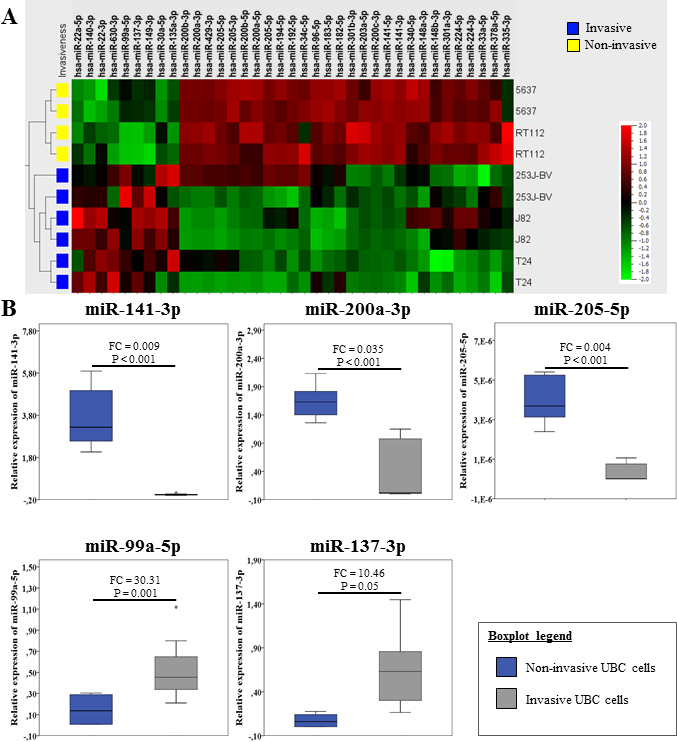
Invasive UBC cells are characterized by a specific miRNA pattern (**A**) The miRNA expression levels in invasive UBC cells (blue) compared to non-invasive UBC cells (yellow). Unsupervised hierarchical cluster analysis of differentially expressed miRNAs. *P*-value was determined using Mann–Whitney *U* test. (Biological replicates *n* = 2 per cell type; *p*-value < 0.05; Fold Change > 1.5; σ = 0.2); (**B**) Relative expression of 5 deregulated miRNAs in invasive UBC cells (grey) compared to non-invasive cells (dark blue) quantified by qPCR (biological replicates *n* = 3 per cell line; invasive cells *n* = 9; non-invasive cells *n* = 6). The expression level was normalized by RNU48. *P*-value was determined using Mann–Whitney *U* test. *P* = *p*-value; FC = Fold Change.

The miRNA microarray analysis revealed 37 miRNAs which were significantly differentially expressed (*P* < 0.05; Fold Change (FC) > 1.5) in invasive UBC cells (T24; J82; 253J-BV) compared to non-invasive cells (RT112; 5637). 29 miRNAs revealed a lower expression and 8 miRNAs a higher expression in these cell lines (Figure 1A). Based on the FC of microarray analysis 3 significantly down-regulated (miR-141-3p; -200a-3p; -205-5p) and 2 significantly up-regulated (miR-99a-5p; -137-3p) miRNAs were selected for quantitative validation using real-time PCR (qPCR). We confirmed the microarray data for the 5 miRNAs (*P* < 0.05) (Figure 1B).

### Urothelial carcinoma cells release exosomes

**Figure 2:**
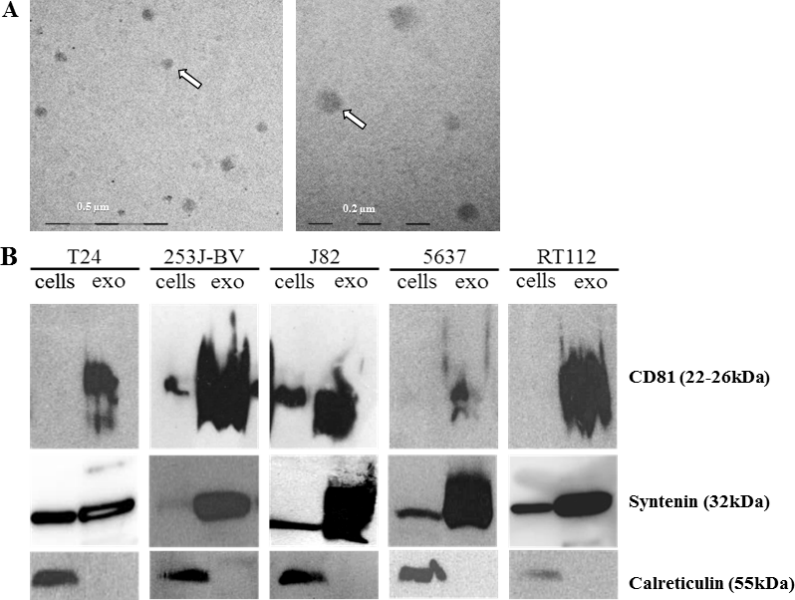
UBC cells secrete exosomes (**A**) Tumor-associated exosomes obtained from T24 cells were isolated after 72 h and analyzed by electron microscopy. T24 exosomes have a mean size of 57 nm with a standard deviation of 16nm. (Scale; 500 nm and 200 nm; white arrows indicate exosomes); (**B**) Western Blot analyses of different UBC cells and their secreted exosomes (exo) for exosomal markers (CD81 (22–26 kDa); syntenin (32 kDa)) and the cellular contamination marker (calreticulin (55 kDa)).

Quantitative and qualitative analyses of exosomes secreted by UBC cell lines were performed by different techniques. Exemplarily electron microscopy of isolated particles from T24 cells verified the typical exosomal morphology and size (median size: 57 nm ± 16 nm standard deviation) (Figure 2A). Size distribution analyses using nanotracking analysis (NTA) revealed the mean exosomal size of 50 to 100 nm (Table 1). The size of T24 exosomes measured by NTA (75 nm ± 13 nm) and electron microscopy (57 nm ± 16 nm) was comparable. The high presence of the exosomal markers syntenin and CD81 and no presence of calreticulin as marker of the endoplasmatic reticulum, confirmed the purity of isolated UBC-secreted exosomes (Figure 2B).

**Table 1:** Number and size of exosomes from invasive and non-invasive UBC cells determined with NTA

Cell line	Invasiveness	Particle size [nm]
T24	Invasive	75 ± 13
253J-BV		81 ± 15
J82		61 ± 19
RT112	Non-invasive	61 ± 23
5637		69 ± 19

Particle size are calculated as median ± standard deviation of 3 biological replicates per cell lines and technical replicates = 3 per biological replicates.

### Exosomes secreted by invasive UBC cells exhibit a specific miRNA expression signature

**Figure 3:**
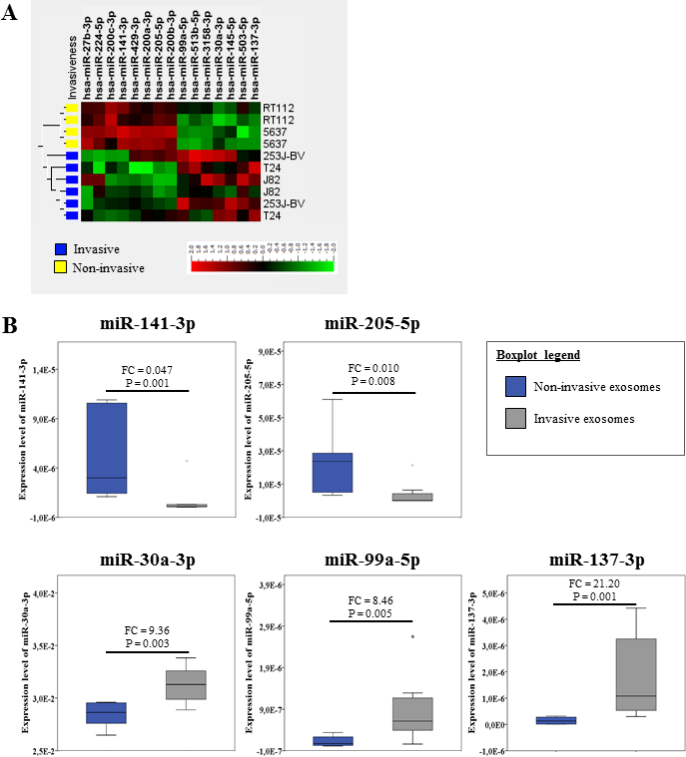
UBC cells produce and secrete exosomes with a specific miRNA expression pattern depending on the invasiveness of parental cells (**A**) The miRNA expression levels in exosomes secreted by invasive UBC cells (blue) compared to exosomes obtained from non-invasive UBC cells (yellow). Unsupervised hierarchical cluster analysis of differentially expressed miRNAs. *P*-value was determined using Mann–Whitney *U* test. (Biological replicates *n* = 2 per cell type; *p*-value < 0.05; Fold Change > 1.5; σ = 0.2); (**B**) Expression level of 5 deregulated miRNAs in exosomes secreted by invasive UBC cells (grey) compared to exosomes from non-invasive cells (dark blue). *P*-value was determined using Mann–Whitney *U* test. (Biological replicates *n* = 3 per cell line; invasive cells *n* = 9; non-invasive cells *n* = 6); P = *p*-value; FC = Fold Change.

We identified 15 miRNAs (*P* < 0.05; FC > 1.5) using microarrays, which were significantly differently expressed in exosomes secreted by invasive compared to non-invasive UBC cells (Figure 3A). Therefrom, 7 miRNAs were higher and 8 lower expressed in exosomes of invasive cells. Based on these results we selected 3 up-regulated (miR-30a-3p; -99a-5p; -137-3p) and 5 down-regulated (miR-27b-3p; -141-3p; -145-5p; -200a-3p; -205-5p) exosomal miRNAs of invasive UBC cells for validation by qPCR (Figure 3B). The expression differences of 5 miRNAs (miR-30a-3p; -99a-5p; -137-3p; -141-3p; -205-5p) were verified (*P* < 0.05). For miR-27b-3p (*P* = 0.776), -145-5p (*P* = 0.864) and -200a-3p (*P* = 0.456) qPCR revealed no significant expression differences.

### MiRNAs are selectively packaged into exosomes depending on the invasiveness of parental cells

The miRNA expression profile of parental cells is reflected in part in their exosomes. Based on the microarray analysis 81% of miRNAs detected in invasive UBC cells were also presented in invasive exosomes normalized by using the same amount of RNA. Non-invasive UBC cells share 62% of miRNAs with their released exosomes (data not shown).

**Figure 4:**
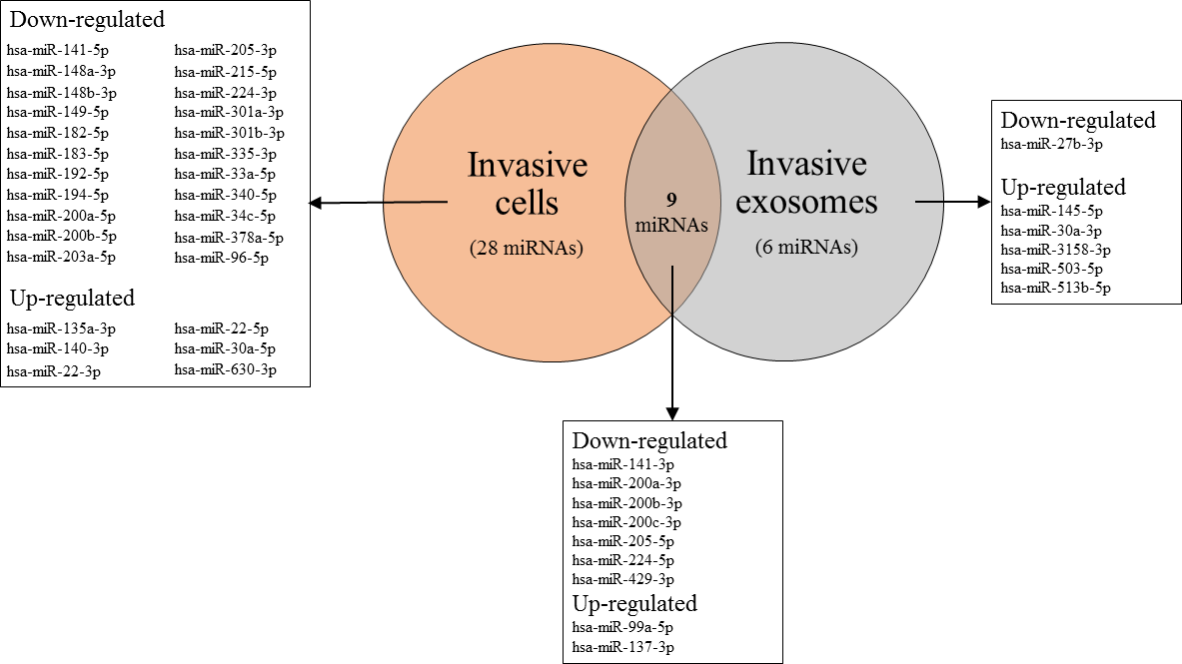
VENN diagram of differentially expressed miRNAs in invasive cells and their exosomes compared to the non-invasive counterparts

Our comprehensive analysis of cellular and exosomal miRNA expression using microarray revealed specific miRNAs which are differently expressed between cells and their associated exosomes. 9 miRNAs including 7 down-regulated (miR-141-3p; -200a-3p; -200b-3p; -200c-3p; 205-5p; -224-5p; -429-3p) and 2 up-regulated (miR-99a-5p; -137-3p) were equally deregulated in both, invasive cells and their secreted exosomes compared to the non-invasive counterparts (Figure 4; Table 2).

**Table 2:** MiRNAs deregulated in both invasive UBC cells and their secreted exosomes analyzed by microarray

MiRNA	Cells Invasive vs. non-invasive				Exosomes Invasive vs. non-invasive			
	*P*-value	*Q*-value	FC	Expression	*P*-value	*Q*-value	FC	Expression
hsa-miR-141-3p	< 0.001	< 0.001	0.001	down	0.01	0.252	0.001	down
hsa-miR-200a-3p	0.01	0.040	0.013	down	0.04	0.999	0.019	down
hsa-miR-200b-3p	0.01	0.044	0.008	down	< 0.001	0.999	0.027	down
hsa-miR-200c-3p	< 0.001	< 0.001	0.001	down	< 0.001	0.428	0.055	down
hsa-miR-205-5p	0.01	0.042	0.002	down	0.04	0.999	0.069	down
hsa-miR-224-5p	0.03	0.119	0.024	down	0.04	0.999	0.071	down
hsa-miR-429-3p	0.02	0.030	0.011	down	0.04	0.999	0.075	down
hsa-miR-99a-5p	0.01	0.100	8.56	up	0.04	0.999	3.83	up
hsa-miR-137-3p	0.01	0.038	41.34	up	0.01	0.999	9.25	up

Invasive cells *n* = 6; non-invasive cells *n* = 4; biological replicates per cell line *n* = 2; *P*-value was determined using Mann–Whitney *U* test. *Q*-value was determined using Student`s *t* test. *P* < 0.05; FC > 1.5; σ = 0.2 (FC = Fold Change).

On the other hand, 28 miRNAs including 22 down-regulated and 6 up-regulated miRNAs were only found to be differently expressed in invasive UBC cells but not in their exosomes (Figure 4; Table 3). Furthermore, 6 miRNAs showed specific alterations in exosomes derived from invasive and non-invasive parental cells, but no differential expression was observed in their parenteral cells. Therefrom, miR-30a-3p was exemplarily verified by qPCR (FC = 9.36; *P* = 0.003; Figures 3B and 4).

**Table 3:** Differentially expressed miRNAs in invasive cells or their exosomes analyzed by microarray

MiRNAs only deregulated in invasive UBC cells				
MiRNA	*P*-value	*Q*-value	FC	Expression
hsa-miR-205-3p	0.01	0.03	0.03	down
hsa-miR-148a-3p	0.02	0.04	0.05	down
hsa-miR-203a-5p	0.01	< 0.001	0.08	down
hsa-miR-224-3p	0.04	0.04	0.09	down
hsa-miR-335-3p	0.04	0.15	0.10	down
hsa-miR-183-5p	0.01	0.01	0.11	down
hsa-miR-141-5p	0.01	< 0.001	0.11	down
hsa-miR-200a-5p	0.01	0.02	0.15	down
hsa-miR-301b-3p	0.01	0.001	0.15	down
hsa-miR-340-5p	0.02	0.04	0.15	down
hsa-miR-149-5p	0.01	< 0.001	0.17	down
hsa-miR-182-5p	0.01	0.04	0.18	down
hsa-miR-96-5p	0.01	0.04	0.17	down
hsa-miR-194-5p	0.02	0.04	0.18	down
hsa-miR-215-5p	0.04	0.04	0.20	down
hsa-miR-192-5p	0.02	0.05	0.21	down
hsa-miR-301a-3p	0.01	0.04	0.22	down
hsa-miR-34c-5p	0.04	0.13	0.24	down
hsa-miR-200b-5p	0.01	0.03	0.24	down
hsa-miR-33a-5p	0.01	0.04	0.26	down
hsa-miR-148b-3p	0.04	0.13	0.29	down
hsa-miR-378a-5p	0.01	0.04	0.29	down
hsa-miR-22-5p	0.02	0.10	3.49	up
hsa-miR-30a-5p	0.04	0.16	3.42	up
hsa-miR-22-3p	0.01	0.04	3.90	up
hsa-miR-135a-3p	0.02	0.14	4.40	up
hsa-miR-140-3p	0.01	0.02	5.25	up
hsa-miR-630-3p	0.02	0.07	5.61	up
**MiRNAs only deregulated in exosomes secreted by invasive UBC cells**				
**MiRNA**	***P*-value**	***Q*-value**	**FC**	**Expression**
hsa-miR-27b-3p	0.04	0.99	0.27	down
hsa-miR-503-5p	0.04	0.99	2.87	up
hsa-miR-513b-5p	0.02	0.99	2.87	up
hsa-miR-145-5p	0.04	0.99	3.38	up
hsa-miR-3158-3p	0.02	0.99	3.39	up
hsa-miR-30a-3p	0.02	0.99	4.27	up

Invasive cells *n* = 6; non-invasive cells *n* = 4; biological replicates per cell line *n* = 2; *P*-value was determined using Mann–Whitney *U* test. *Q*-value was determined using Student's *t* test. *P* < 0.05; FC > 1.5; σ = 0.2 (FC = Fold Change).

### MiRNAs are differently expressed in tumors tissue and urine exosomes of bladder cancer patients

To ensure that our findings have clinical relevance we investigated the *in vitro* results in human patient derived samples. We analyzed expression of selected miRNAs based on microarray analysis for tumor tissues and quantified exosomal miRNAs in urine samples by qPCR analyses (Table 4). Tissue and urine samples were not paired.

**Table 4:** Clinical-pathological parameters of the study cohorts

		Tumor tissue	Urine exosome
		(*n* = 24)	(*n* = 21)
**Age**	Mean/Median	68/68 (48–83)	68/70 (53–83)
**Sex**	Male	17	15
	Female	7	6
**T**	pTa	10	3
	pT1	0	4
	pT2	5	5
	pT3	5	7
	pT4	4	2
**N**	N0	7	7
	N+	5	4
	NX	12	10
**Grade**	G1	4	0
	G2	7	7
	G3	13	14

**Figure 5:**
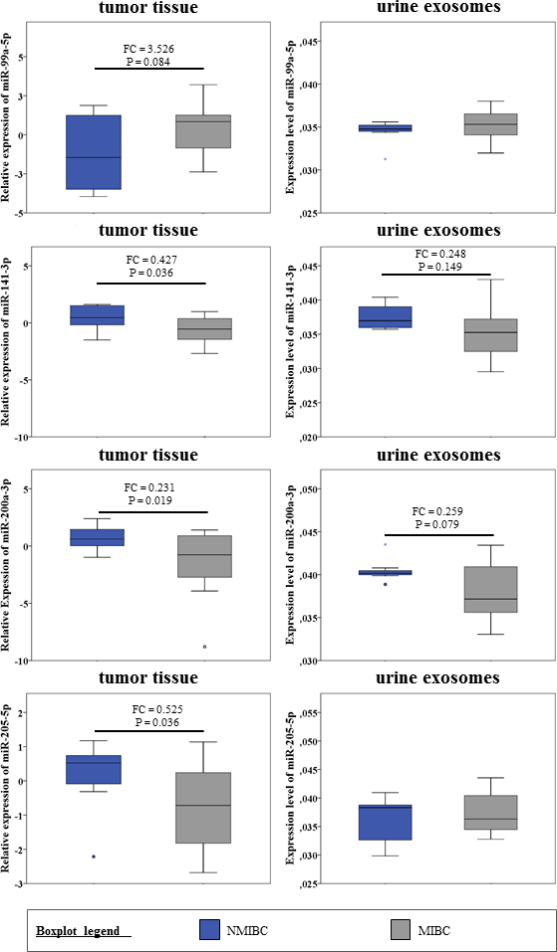
miRNA expression of 4 deregulated miRNAs in primary tumor tissue and urine exosomes of bladder cancer patients Expression levels of miRNAs (miR-99a-5p; -141-3p; -200a-3p; -205-5p) in primary tumor tissue and urine exosomes of MIBC (grey) compared to NMIBC (dark blue). The expression of miRNAs in tissue was quantified by microarray analysis and for urine exosomes by qPCR. *P*-value was determined using Mann–Whitney U test. (Tissue (NMIBC (Ta) *n* = 10; MIBC (≥ T2) *n* = 14; FC > 1.5; σ = 0.2); urine exosomes (NMIBC (Ta, T1) *n* = 7; MIBC (≥ T2) *n* = 14)).

Cellular expression changes of the 5 miRNAs could be partially verified in primary tumors of MIBC compared to NMIBC (Figure 5). MiR-141-3p, -200a-3p and -205-5p were significantly down-regulated in MIBC tumors (*P* < 0.05) compared to NMIBC. For miR-99a-5p we detected in trend an up-regulation in MIBC (*P* = 0.084). MiR-137-3p revealed no significant expression differences between MIBC and NMIBC.

Furthermore, the expression of 8 miRNAs was measured in urine exosomes of MIBC and NMIBC patients using qPCR (Figure 5). MiRNA miR-137-3p was not detectable in urine exosomes. For miR-200a-3p we revealed in trend (*P* = 0.079) a down-regulation in urine exosomes of MIBC. The remaining 6 miRNAs (miR-27b-3p; -30a-3p; -99a-5p; 141-3p; -145-5p; -205-5p) showed no expression differences between MIBC and NMIBC patients.

## DISCUSSION

MIBC are characterized by specific molecular alterations, such as p53 and retinoblastoma (Rb) mutation, which are associated with high aggressiveness and poor prognosis. It is currently unknown how MIBC can induce a tumor-promoting microenvironment by extracellular trafficking of molecules influencing the surrounding tissue. Recent studies have shown that tumor cells release exosomes with specific miRNAs which may stimulate several tumor-promoting processes such as angiogenesis and formation of a premetastatic niche [19, 22]. Furthermore, based on their stability in body fluids, especially exosomal miRNAs are discussed to be useful diagnostic and prognostic biomarkers in liquid biopsies.

In this study we compared *in vitro* the cellular and exosomal miRNA expression in UBC cell lines with different invasive potential. For the first time we demonstrated that miRNAs are not only differently expressed in invasive cells of UBC but also in their exosomes compared to the non-invasive counterparts. These results confirmed the hypothesis that the molecular content of exosomes is, at least in part, similar to that of host cells and reflects their cellular properties. In previous studies some of the identified miRNAs have already been described as potential tissue based markers for MIBC [23–25]. The most characteristic miRNAs are members of the miR-200 family (miR-141-3p/5p; -200a/b/c-3p/5p) and miR-205-3p/5p, which are related to the epithelial to mesenchymal transition (EMT)-associated phenotype with a high ability of invasiveness. Dyrskjøt et al. and Pignot et al. have demonstrated a correlation between the down-regulation of members of miR-200 family and high grade or MIBC tumors [26, 27]. The down-regulation of miR-200 family members and miR-205-3p/5p are crucial in UBC carcinogenesis through promoting EMT by targeting ZEB1 and ZEB2. Loss of the miR-200 family leads to a decrease in epithelial proteins and enhanced expression of mesenchymal proteins, which results in a loss of cell adhesion and an increased invasiveness [25, 28–30]. Furthermore, the majority of MIBC comparing to NMIBC exhibited an up-regulation of miR-99a, -100 indicating that these miRNAs may have an oncogenic effect in invasive UBC [20, 31, 32].

In addition, we tested the *in vitro* deregulated miRNAs in tumor tissue and urine samples of bladder cancer patients in order to proof the clinical and biological relevance. We could show that the cell culture system reflects the invasion-associated miRNA pattern of tumor samples. However, urinary exosomes exhibit only in part the miRNA alterations detected in cell line exosomes. This was expected since in urine exosomes secreted by different cell types are present and not pure tumor cell exosomes as in cell culture supernatants. Even tumor cells release significantly elevated levels of exosomes, the wide range of exosomes in urine could disguise the tumor cell related differences of exosomal miRNA expression. In order to identify reliable exosome based biomarkers, which was not the focus of the present study, alternative approaches should be used including: 1. Development of exosome isolation techniques based on tumor cell surface markers, 2. starting with screening in liquid biopsies but not based on selected tissue or *in vitro* putative candidates.

To the best of our knowledge studies analyzing miRNAs as diagnostic markers for the discrimination between MIBC and NMIBC in liquid biopsies are not published. Nevertheless, previous studies based on general expression screening methods in liquid biopsies have indicated the possibility to use miRNAs as a marker for detection of UBC. MiRNAs (including miR-21-5p; -100; -200 family; -205-3p/5p) isolated from urine, plasma and serum of bladder cancer patients are differently expressed compared to healthy controls [33–39]. In general, exosomes from liquid biopsies might be a more appropriate source for biomarker research in MIBC than tissue sections, since the content of exosomes seem to reflect the heterogeneity of primary tumors better than a single tissue samples or biopsies [40, 41].

Furthermore, our results support the hypothesis that some miRNAs are selectively packed into exosomes. We found miRNAs like miR-30a-3p, deregulated only in exosomes of invasive UBC cells compared to non-invasive counterparts, but not in their parental cells. The underlying mechanisms of different miRNA profiles in exosomes and their parental cells are subject of current research. Previous studies have assumed a passive loading of miRNAs into exosomes, but most recent reports have demonstrated that miRNAs are selectively packaged into exosomes by specific proteins, such as heterogeneous nuclear ribonucleoprotein (hnRNP), or by binding to specific sequences on the 3´end of the miRNA [42–44]. Due to the selective secretion of miRNAs into exosomes tumor cells can rapidly regulate their intracellular miRNA level. Thereby, tumor cells generate a tumor-promoting phenotype by self-protection the cell against tumor-suppressive miRNAs [45]. Furthermore, it has been shown that tumor-associated exosomal miRNAs were taken up by recipient cells in the TME, such as immune cells, endothelial cells and fibroblasts. The uptake leads to a shift of their intracellular gene expression level, which could result in the generation of a tumor-promoting and most notably invasion-promoting TME [18, 46–49]. Finally, the possible role of exosomal miRNAs in bladder tumors still needs to be identified.

So far only few studies on UBC are published, which investigated the role of exosomes in the interaction between UBC cells and the microenvironment. Exosomes from cell lines as well as urine derived exosomes from high grade UBC patients promote migration of endothelial cells. Angiogenesis was stimulated inducing a tumor-promoting environment by an efficient nutrition supply [50]. Furthermore, Franzen et al. have shown that tumor-associated exosomes of invasive UBC cells and urine exosomes trigger EMT formation of normal urothelial cells, which can also support the tumorigenesis [10, 51]. In addition to the effects on cells in the TME, the data of Yang et al. have revealed an self-stimulating effect of UBC exosomes on cell viability of the parental UBC cells by reducing apoptosis via Akt/ERK pathway in a time- and dose-dependent manner [52]. It seems very likely that exosomal miRNAs are also involved in these processes.

A limitation of this study is the missing normalization of the exosomal miRNA expression which is still a matter of controversial discussions. At the annual meeting of the International Society of Extracellular Vesicles (ISEV) in Rotterdam 2016 it was pointed out that at present the normalization of miRNA expression of extracellular vesicles including exosomes is a major challenge, because no reliable controls are available. Well-established normalization procedures applied for tissues or cell cultures like the inclusion of internal or external reference genes are not applicable to exosomes, because the exosomal content of the reference genes (e. g. RNU48) varies. Therefore, it is important to develop more effective strategies.

In summary, we demonstrated *in vitro* that UBC derived exosomes are characterized by a specific miRNA signature depending on the invasiveness of the parental cells. The identified miRNAs reflect in part the altered expression changes of the parental UBC cells. Whether and to what extend these miRNAs regulate intercellular communication between tumor cells and their TME has to be analyzed in further studies. The extensive knowledge about the molecular pathways could lead to a better understanding of the complexity of carcinogenesis in MIBC, resulting in new individual treatment strategies. Furthermore, it has to be proven if these invasion-associated exosomal miRNA alterations may be used as possible liquid biomarkers for the discrimination between NMIBC and MIBC.

## MATERIALS AND METHODS

### Cell culture

For our study we used 5 different UBC cell lines. RT112 cell line (DSMZ; Germany) is established from a low grade (G2) transitional cell carcinoma (TCC) from a woman (age unknown) in 1973 and were cultured RPMI-1640 medium (Sigma Aldrich; United States) supplemented with 10% fetal bovine serum (FBS; Sigma Aldrich; United States). 5637 (DSMZ; Germany) were originated from a primary low grade (G2) TCC of a 68-year old man in 1974 and cultured in RPMI + 10% FBS. Cell line T24 (DSMZ; Germany) was established from a high grade (G3) TCC from a 81-year-old woman in 1970 and in maintained Dulbecco`s Modified eagle medium (DMEM; Sigma Aldrich; United States) + 10% FBS. J82 (ATCC; United States) was isolated from a primary high grade (G3) TCC of a 58-year-old man in 1974 and cultured in DMEM + 10% FBS. 253J-BV (provided generously by Dr. Arshish Kamat, MD Anderson Cancer Center; United States) was derived following 5 serial passages of parental cell line 253J through the bladder of an athymic nude mice [53]. The parental cell line 253J was originated from lymph node metastasis of a high grade (G4) and T4 TCC tumor in 1972 resected from a 53-year-old man [54–56]. The cultivation of this cell line requires DMEM + 10% FBS. All cells were grown at 37°C, in 5% CO_2_ in a humidified atmosphere, and harvested for analysis at 80–90% confluence. For all cell lines a current authentication is available.

### Patient material

Bladder tumor samples (*n* = 24) were obtained from patients undergoing transurethral bladder resection (TUR) or radical cystectomy at the Department of Urology and Pediatric Urology in Homburg (Saarland University Medical Center). The study was approved by the Saarland ethic committee. All patients signed a written informed consent. Samples were routinely fixed in formalin and classified by pathologist of the Institute of Pathology (Saarland University Medical Center) (Table 4). Tumor stage and grade was determined according to the WHO criteria and TNM classification. Urine supernatant samples (*n* = 21) collected between 2012 and 2016 (Table 4) were stored at −80°C. All tumors were classified in MIBC (≥ T2) and NMIBC (Ta, T1) according to the current TNM classification. Tissue and urine samples were not paired.

### Exosome purification

For exosome isolation the cell culture medium was replaced by normal medium supplemented with exosome-free FBS. Exosome-free FBS was generated by ultracentrifugation at 200,000 g for minimum 18 h. The cell were incubated over 24–72 h. Cell culture supernatants were centrifuged by different centrifugation steps. The first three steps (400 g (10 min); 2,000 g (30 min) and 15,000 g (30 min)) were performed to remove residual cells and cellular debris as well as apoptotic bodies and microparticles. Exosome isolation was performed using a commercial kit from Life Technologies according to the manufacture instructions. For further experiments the pellet was dissolved in PBS (Sigma-Aldrich; United Stated) or directly lysed by lysis buffer (50 mM Tris; 150 mM NaCl; 1 mM MgCl_2;_ 1% Triton X-100).

Urine exosomes were isolated with an adapted protocol using the kit from Life Technologies. First, urine supernatant was centrifuged by differential centrifugation (2,000 g (30 min); 15,000 g (30 min)). Second, the supernatant was mixed in a ratio 1:1 with the total exosome isolation reagent or urine (Life Technologies; United States). Following steps were performed according to the manufactures protocol. For further experiments the pellet was lysed in 700 μl QIAzol Lysis Reagent (Qiagen, Germany).

### Nanoparticle tracking analysis

The size and concentration of vesicles was analyzed by NanoSight LM10 system and NTA software v2.3 (NanoSight Ltd; United Kingdom). Video recordings of 60 s and approximately 500–1,200 tracks were analyzed per sample. Particle size are calculated as median ± standard deviation of 3 biological replicates per cell lines and 3 technical replicates per biological replicates were captured.

### Electron microscopy

Exosome pellets were fixed with 2% paraformaldehyde. Afterwards, exosomes were placed on Formvar-carbon coated EM grid. Grid was washed in aqua dest. and fixed again with 1% glutaraldehyde. After washing in aqua dest. the grid was incubated in uranyl-oxalate solution, pH 7. The grid was washed in aqua dest. and dried. Transmission electron microscopy (TEM; Carl Zeiss GmbH; Germany) was performed at 100 kV and electron micrographs were captured with TEM software (Carl Zeiss GmbH; Germany).

### Western blot analysis

Total cell and exosome lysates were prepared in a lysis buffer in the presence of a Protease inhibitor Mix M (1:100; Serva; Germany). Protein concentration was measured using Pierce™ BCA Protein Assay Kit (Thermo Fisher; United States). Exosome and cell lysates were mixed with loading buffer with and without DTT. The proteins were separated by a 12.5% SDS-PAGE and transferred to a PVDF membrane (Pall Life Science; Germany). Membranes were blocked in 5% non-fat milk powder (Sigma Aldrich; United States) in 1x TBS; containing 0.1% Tween 20 (TBS-T) or NETG solution (50 mM Tris; 150 mM NaCl; 5 mM EDTA; 0.05% Tween 20; 0.04% gelatin; pH 7.5). Immunoblotting of exosomal and cellular protein content was performed at 4°C overnight using antibodies against CD81 (1:500; Santa Cruz; United States), syntenin (1:1,000; Abcam; United Kingdom), calreticulin (1:1,000; Cell Signaling; United States) and GAPDH (1:1,000; Cell Signaling; United States). After incubation with primary antibodies the membranes were washed thrice in 1× TBS-T 20 for 5 min and incubated for 1 h at room temperature with horseradish peroxidase (HRP) conjugated goat anti-mouse IgG (1:10,000; Dianova; Germany) or anti-rabbit IgG (1:1,000; Cell Signaling; United States) in blocking solution. Membranes were washed twice for 5 min in 1 × TBS-T. Immunreactive bands were visualized using Luminata Classico Western HRP (Millipore; Germany) and detected with software Fusion FX7 of the ECL reader Fusion SL VilberLourmat (PeqLab; Germany).

### Isolation of miRNAs

TotalRNA was isolated from cells and exosomes using miRNeasy Mini Kit (Qiagen; Germany). Previously, free-circulating RNA was removed by resuspending the exosome pellet in 100 μl PBS and treatment with 10 units RNase ONE™ Ribonuclease (Promega; United States) for 30 min at room temperature. RNase reaction was stopped by adding 10 units RiboLockRNAse Inhibitor (Thermo Fisher Scientific; United States) to the mixture and incubation for 10 min at room temperature. Afterwards the isolation was performed according to a modified manufacture protocol. RNA concentration was measured by NanoDrop 1000 (Thermo Fisher Scientific; United States). MiRNA of urine exosomes was isolated according to the protocol of cells and *in vitro* exosomes.

TotalRNA of formalin-fixed paraffin-embedded (FFPE) primary bladder tumors was isolated using miRNeasy FFPE Kit (Qiagen, Germany). 20 sections of each tumor were prepared on a microscope slides to perform a macro-dissection of tumor areas. Tumor sections were transferred into a reaction tube. Afterwards, isolation of totalRNA was performed according to the manufacture protocol and RNA concentration was measured by NanoDrop 1000 (Thermo Fisher Scientific; United States).

### MiRNA expression analyses

The miRNA expression analyses were performed using human miRNA microarray (Agilent Technologies; version 16; United States) on 5 UBC cell lines and their secreted exosomes. The samples were prepared as biological duplicates. Total RNA (100 ng) was labeled and hybridized on a microarray (miRNA complete labeling and hybridization kit) and afterwards scanned using DNA Microarray Scanner (Agilent Technologies; United States). All steps were performed according to the manufactures protocol. After extraction of raw data using Feature Extraction Software (Agilent Technologies; United States) data were analyzed using Qlucore software (version 3; Qlucore; Sweden). The total gene signal was normalized to the 75th percentile of signal intensity.

For validation of microarray results, a qPCR was performed in triplicates for each UBC cell line and their secreted exosomes and for urine exosomes. First, total RNA (50 ng for cells and their exosomes; 10 ng for urine exosomes) was reverse-transcribed using specific primers (miR-27b-3p; -30a-5p; -99a-5p; -137; -141-3p; -145-5p; -200a-3p; -205-5p and endogenous control for cells RNU48) and TaqMan MicroRNA Reverse Transcription Kit (Life Technologies; United States) according to the manufactures protocol. We selected the endogenous control RNU48 for normalization of miRNA expression levels. Our previous data have shown that RNU48 is high stable expressed between the different UBC cells. Afterwards, cDNA transcribed from urine exosomes were preamplified with TaqMan PreAmp Master Mix (Applied Biosystems, United States) and TaqMan MicroRNA Assays (Applied Biosystems, United States). The PreAmp product was diluted 1:5 in TE buffer (Applied Biosystems, United States). qPCR was performed in triplicates using specific TaqMan primers and TaqMan Gene Expression Master Mix (Life Technologies; United States) in a StepOnePlus™ System (Life Technolgies; United States). Average was calculated and expression values of cells were normalized using control RNU48. At the moment, no suitable normalization methods of exosomal miRNAs are available. Therefore, the miRNA expression of exosomes are normalized on quantity of RNA (100 ng) used for qPCR.

### Statistical analyses

Analyses of the microarray data were performed using Student`s *t* test and unsupervised hierarchical clustering unpaired (two-group comparison) with the help of Qlucore software (version 3; Qlucore; Sweden) set to the parameters FC > 1.5; *p* > 0.05 and variance σ = 0.2. The q-value was determined by Student`s t test using Qlucore software (version 3; Qlucore; Sweden). Non-parametric Mann–Whitney U test with the software IBM SPSS Statistics 20 (IBM; United States) was used to analyze the raw data of microarrays. QPCR data were analyzed using REST 2009 software (Version 2009; developed by M. Pfaffl (Technical University Munich; Germany) and Qiagen; Germany) and non-parametric Mann–Whitney U test using IBM SPSS Statistics 20 (IBM; United States).

## Abbreviations

Akt: Proteinkinase B
BCA: Bicinchoninic acid
DMEM: Dulbecco`s Modified eagle medium
DTT: Dithiothreitol
EMT: Epithelial to mesenchymal transition
ERK: Extracellular signal-regulated kinases
Exo: Exosome
FBS: Fetal bovine serum
FC: Fold Change
FFPE: Formalin-fixed paraffin-embedded
hnRNP: Heterogeneous nuclear ribonucleoprotein
HRP: Horseradish peroxidase
ISEV: International Society of Extracellular Vesicles
MIBC: Muscle-invasive bladder cancer
miRNA: MicroRNA
NMIBC: Non-muscle-invasive bladder cancer
NTA: Nanotracking analysis
PBS: Phosphate buffered saline
qPCR: Quantitative polymerase chain reaction
Rb: Retinoblastoma
SDS-PAGE: Sodium dodecyl sulfate polyacrylamide gel electrophoresis
TBS-T: Tris buffered saline TWEEN
TCC: Transitional cell carcinoma
TEM: Transmission electron microscopy
TME: Tumor microenvironment
TUR: Transurethral bladder resection
UBC: Urinary bladder cancer
ZEB1/2: Zinc Finger E-box Binding Homeobox 1/2.
